# Chronic administration of anticonvulsants but not antidepressants impairs bone strength: clinical implications

**DOI:** 10.1038/tp.2015.38

**Published:** 2015-06-02

**Authors:** P W Gold, M G Pavlatou, D Michelson, C M Mouro, M A Kling, M-L Wong, J Licinio, S A Goldstein

**Affiliations:** 1 Clinical Neuroendocrinology Branch, National Institute of Mental Health, National Institutes of Health, Bethesda, MD, USA; 2 Merck, Whitehouse Station, NJ, USA; 3 Orthopaedic Research Laboratories, University of Michigan, Ann Arbor, MI, USA; 4 Behavioral Health Service, Philadelphia VA Medical Center, Philadelphia, PA, USA; 5 Department of Psychiatry, University of Pennsylvania School of Medicine, Philadelphia, PA, USA; 6 Department of Translational Medicine, John Curtin School of Medical Research, Australian National University, Canberra, ACT, Australia

## Abstract

Major depression and bipolar disorder are associated with decreased bone mineral density (BMD). Antidepressants such as imipramine (IMIP) and specific serotonin reuptake inhibitors (SSRIs) have been implicated in reduced BMD and/or fracture in older depressed patients. Moreover, anticonvulsants such as valproate (VAL) and carbamazepine (CBZ) are also known to increase fracture rates. Although BMD is a predictor of susceptibility to fracture, bone strength is a more sensitive predictor. We measured mechanical and geometrical properties of bone in 68 male Sprague Dawley rats on IMIP, fluoxetine (FLX), VAL, CBZ, CBZ vehicle and saline (SAL), given intraperitoneally daily for 8 weeks. Distinct regions were tested to failure by four-point bending, whereas load displacement was used to determine stiffness. The left femurs were scanned in a MicroCT system to calculate mid-diaphyseal moments of inertia. None of these parameters were affected by antidepressants. However, VAL resulted in a significant decrease in stiffness and a reduction in yield, and CBZ induced a decrease in stiffness. Only CBZ induced alterations in mechanical properties that were accompanied by significant geometrical changes. These data reveal that chronic antidepressant treatment does not reduce bone strength, in contrast to chronic anticonvulsant treatment. Thus, decreased BMD and increased fracture rates in older patients on antidepressants are more likely to represent factors intrinsic to depression that weaken bone rather than antidepressants *per se*. Patients with affective illness on anticonvulsants may be at particularly high risk for fracture, especially as they grow older, as bone strength falls progressively with age.

## Introduction

Recent data in large populations of older individuals indicate that specific serotonin reuptake inhibitors (SSRIs) are associated with the risk of decreased bone mineral density (BMD) and/or hip fracture.^1, 2, 3^ Other studies report that both SSRIs and tricyclic antidepressants (TCAs) increase the risk of hip fracture in older patients without commenting on BMD.^4, 5, 6^ The impact of antidepressants on BMD is complicated by the fact that many papers also find that major depression is associated with loss of BMD *per se.*^7, 8, 9^ This loss of BMD in patients with major depression potentially reflects a number of hormonal alterations that may be risk factors for decreased BMD, including hypercortisolism, decreased secretion of growth hormone and hypothalamic hypogonadism.^10, 11, 12^ In addition, melancholic patients with major depression have around-the-clock increases in plasma and CSF norepinephrine secretion,^13^ and recent data indicate that sympathetic drive contributes to bone loss.^14^ Depressed patients are also in a mild proinflammatory state, characterized by changes such as increased plasma IL-6^15^ and acute phase protein levels.^16^ It is well known that inflammation has a major role in some forms of osteoporotic bone loss,^17^ especially at the hip. In our premenopausal women with major depression, bone loss was considerably greater at the hip than at the spine,^7^ in contrast to the usual pattern of greater loss of vertebral bone in common forms of osteoporosis. This pattern is more compatible with inflammatory causes of osteoporosis. Thus, inflammation may be particularly important in the bone loss of depressive illness.

Anticonvulsants are also well known to contribute to reductions in BMD and increase the rate of bone fracture. These compounds are used not only to treat epilepsy, but also major depressive illness as well. Several factors may contribute to the increased fracture risk reported in patients with epilepsy. Epileptic seizures may *per se* lead to injury. In addition, subjects with epilepsy, especially if children, may have insufficient dietary intake of vitamin D, limited sun exposure, and decreased ambulation and physical activity, all nonspecific factors that can lead to impaired bone structure and function.^18^ However, anticonvulsants may also affect bone mass by specific mechanisms. Their mechanisms include impaired calcium absorption, induction of microsomal enzymes with consequent accelerated hepatic catabolism of vitamin D, secondary hypoparathyroidism, and osteomalacia.^19, 20, 21^

There is extensive and long-term clinical use of antidepressant and antiepileptic agents, and drug-induced osteoporosis is a preventable condition. We thus decided to test whether chronic administration of antidepressants and anticonvulsants to rats may affect mechanical strength, an extremely important determinant of fracture risk in low impact trauma. We know several mechanisms by which anticonvulsants can contribute to low BMD or bone fracture, but knowing more about their impact on specific parameters of bone strength may help contribute to methods of reducing the risk to bone health posed by anticonvulsants.

None of the factors promoting bone loss attributable to anticonvulsants have yet been found as a consequence of antidepressant treatment. Showing whether or not antidepressants decrease bone strength could help clarify the extent to which low BMD or fracture in depression can be, in part, attributable to medication, or is an intrinsic component of depressive illness. Bone strength may be more important than BMD in conferring susceptibility to bone fracture, so that BMD and bone strength are not necessarily synonymous. Therefore, it is of substantial clinical importance to establish whether and how chronic administration of antidepressants to the rat directly affects bone strength.

## Materials and methods

### Drug treatment

Sixty-eight male Sprague Dawley rats were treated either with the antidepressants imipramine (IMIP) or fluoxetine (FLX); with the anticonvulsants valproate (VAL) or carbamazepine (CBZ); or given a sham treatment via daily intraperitoneal injections for 8 weeks. A saline (SAL) sham treatment was used as the control for the IMIP, FLX and VAL groups, while carbamazepine vehicle (CBZ-V) was used as the control for the CBZ group. The FLX group was administered 1 mg kg^−1^ body weight of fluoxetine; the IMIP group was given 5 mg kg^−1^ of IMIP; the VAL group received 250 mg kg^−1^ of valproate, all in 0.5 ml saline vehicle. The SAL group was given 0.5 ml saline alone. The CBZ group was treated with 40 mg kg^−1^ body weight of CBZ in a 1-ml propylene glycol/alcohol/saline vehicle. The CBZ-V group received a sham treatment of 1 ml of CBZ-V alone.

### Mechanical testing

**Figure 1:**
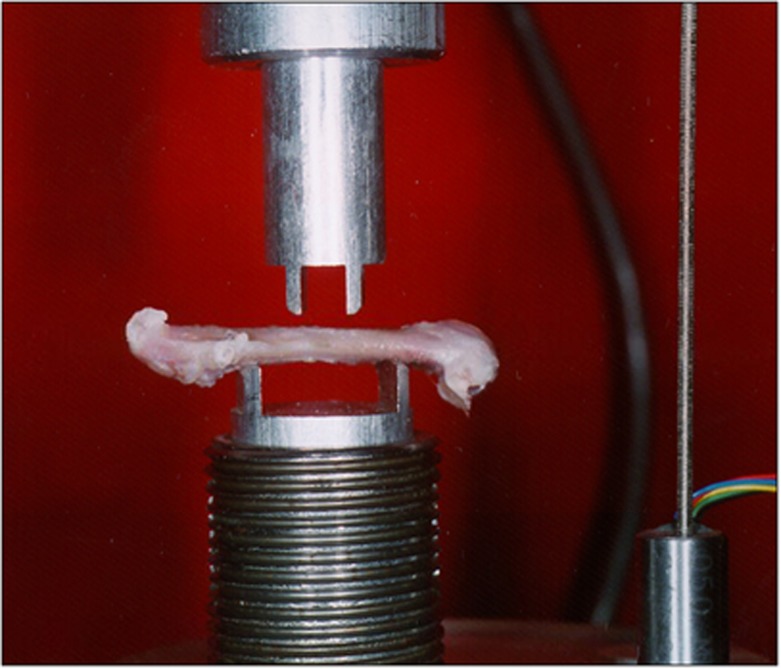
The femurs were loaded to failure by a custom designed four-point bending fixture secured within the structure of an MTS materials testing system. The region of the mid-diaphysis subjected to load between the four loading points corresponded precisely to the volume of bone evaluated by MicroCT.

The right femurs from 53 rats, which had been stored fresh-frozen, were tested to failure in four-point bending using an MTS servohydraulic testing machine (Minneapolis, MN, USA) at a constant displacement of rate of 5 mm s^−1^ (see Figure 1). Load-displacement data were acquired and used to determine loads and displacements to both yield and failure. Stiffness was calculated as the slope of the linear portion of the load-displacement curve.

### Geometrical analysis

The left femurs from 68 rats were scanned in a MicroCT system, and three-dimensional digital images were reconstructed at a resolution of 50 μm. The images were thresholded to distinguish bone from non-bone voxels, and the analysis region was defined as the mid-50% region of the bone (mid-diaphysis). Cross-sectional area, cortical thickness and moments of inertia were determined for each slice, and then averaged along the length of the analysis region. Moment of inertia is a mathematical representation of the distribution of bone material away from the neutral bending axis of the bone, and is directly related to mechanical properties in bending (Please see Appendix 1 for a further explication of this term).

### Statistical analysis

A one-way analysis of variance was used to compare the FLX, IMIP and VAL groups with the SAL group, and to compare the CBZ and CBZ-V groups. Tukey's *post hoc* test was used for comparisons between groups with *P*<0.05 considered statistically significant.

## Results

**Figure 2:**
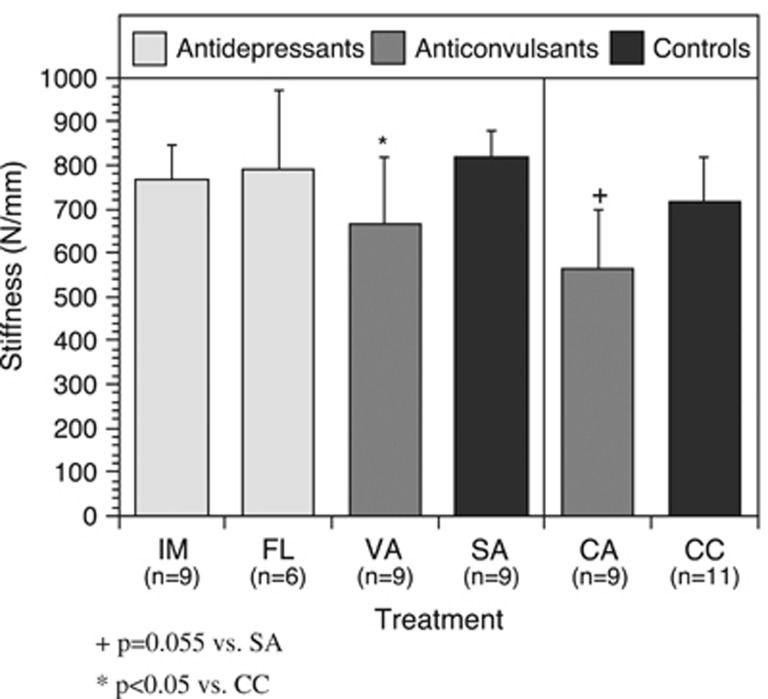
Anticonvulsant drug therapy demonstrated a significant decrease in whole bone stiffness. ^+^*P*=0.055 vs SA. ^*^P<0.05 vs CC. CA, carbamazepine; CC, controls; FL, fluoxetine; IM, imipramine; SA, saline; VA, valproate.

Three months of daily treatment with IMIP and FLX had no impact on stiffness, bone yield or geometric properties of bone consisting of moment of inertia, cortical thickness and cross-sectional area (Figure 2).

**Figure 3:**
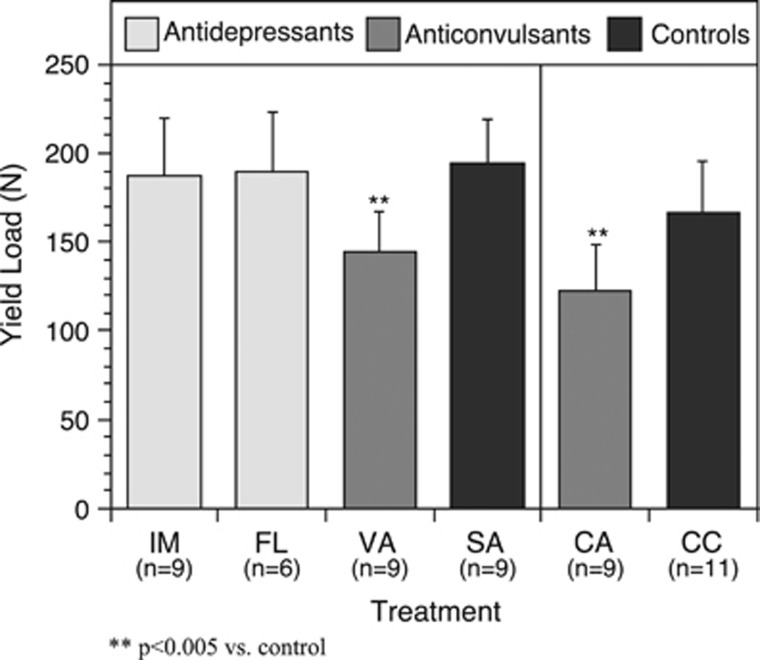
Similar to the results for stiffness, the anticonvulsants significantly reduced the load to yield in femurs. ^**^P<0.005 vs control. CA, carbamazepine; CC, controls; FL, fluoxetine; IM, imipramine; SA, saline; VA, valproate.

In general, anticonvulsant drug therapy causes a decrease in mechanical properties of whole bones. Both the VAL and CBZ groups exhibited significant decreases in stiffness and yield load (see Figures 2 and 3). The VAL group exhibited an 18.5% reduction in stiffness and a 25.3% reduction in yield load when compared with the SAL group. Treatment with CBZ resulted in a 21.3% decrease in stiffness and 26.5% decrease in yield load when compared with the CBZ-V group. Similar trends were seen in failure load results, although the comparison was only statistically significant between the CBZ and CBZ-V groups.

**Figure 4:**
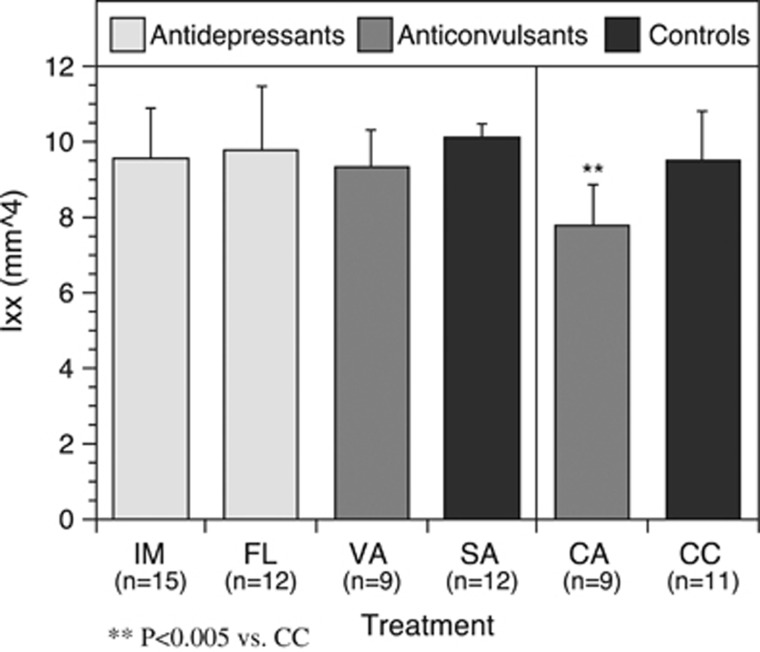
Only the anticonvulsant CBZ demonstrated a significant reduction in the moment of inertia, reflecting a change in the cross-sectional geometry. ^**^P<0.005 vs CC. CA, carbamazepine; CC, controls; FL, fluoxetine; IM, imipramine; SA, saline; VA, valproate.

These alterations in mechanical properties were accompanied by significant geometrical changes only in the CBZ group (see Table 1). Cross-sectional area, moment of inertia and cortical thickness were significantly decreased in the CBZ group when compared with the CBZ-V group. The CBZ group exhibited an 18.3% reduction in moment of inertia when compared with the CBZ-V group, whereas the VAL group only showed a 7.7% decrease when compared with the SAL group (see Figure 4).

## Discussion

Eight-week-old male Sprague Dawley rats were treated either with the antidepressants, IMIP or FLX; anticonvulsants, VAL or CBZ; or given a sham treatment, saline SAL or CBZ vehicle via daily intraperitoneal injections for 8 weeks. The right femur was tested to failure in four-point bending. Load-displacement data were used to determine stiffness, and loads and displacements to both yield and failure. The left femurs were scanned in a MicroCT system to calculate cross-sectional area, cortical thickness and moments of inertia of the mid-diaphyseal region. Neither IMIP nor FLX had any significant effects on cross-sectional area, moment of inertia and cortical thickness. On the other hand, both anticonvulsants adversely influenced multiple determinants of bone strength.

### SSRIs and TCAs: putative effects on BMD and hip fracture

Diem *et al.*^1^ studied 2722 older women prospectively over a 5-year period and concluded that SSRIs led to a significant reduction in BMD at the hip. This finding could be complicated by the fact that patients with depressive illness may lose bone faster than matched controls. Moreover, its functional significance is not clear as loss of BMD does not always correlate precisely with bone strength of relevance to the liability to fracture (*vide infra*).

Haney *et al.*^2^ studied 5995 men 65 years and older cross-sectionally, showing that SSRIs were also associated with significant losses of BMD at the hip and spine, while subjects on TCAs or trazadone had no loss of BMD. Richards *et al.*^3^ studied 5008 community dwelling adults age 50 and above prospectively over a period of five years and also showed a clinically-relevant association between SSRI use, BMD loss at the hip, and a trend for BMD loss at the spine. SSRI treatment was also associated with twofold increased incidence of fragility fracture greater at the hip than at the spine.

Three studies have suggested that both SSRIs and TCAs are associated with increased risk of hip fracture. Liu *et al.*^5^ studied a group of 8239 patients aged 66 years and older and found a twofold increase in hip fracture for both drugs. Similarly, Ziere *et al.*^6^ studied 1289 elderly subjects and found a greater than twofold increase in hip fracture for both drugs. None of these studies examined measures of BMD.

### Bone has functional serotonergic system and responds to norepinephrine as well

*In vitro* and *in vivo* data in experimental animals indicate a functioning serotonin system in bone. Osteoblast and osteoclasts both express a serotonin transporter system, with mechanisms for responding to and taking up serotonin.^22^ Functional receptors for serotonin and the serotonin transporter have been identified in osteoblasts, osteoclasts and osteocytes. Serotonin has been shown to induce murine osteocytes. Serotonin and human osteoclast differentiation *in vitro*. The SSRI FLX inhibits osteoblast differentiation and osteoclast differentiation,^23^ effects that would have opposing effects on BMD.

Serotonin transporter (5HTT) knockout is associated with decreased bone accrual during growth. The authors suggest that this might relate to an effect in the 5HTT in reducing skeletal responsiveness to mechanical loading. However, using the established loading model, 5HTT−/− and 5HTT+/+ mice were exposed to identical osteogenic stimuli, no influence of the null mutation was found on skeletal growth.

Activation of the sympathetic nervous system promotes bone resorption.^14, 24^ Yirmiya *et al.*^25^ found that sympathetic nervous system activation in response to behavioral stressors causes bone loss in association with behavioral features of depression. A tricyclic mediated increase in bone fracture is not necessarily compatible with the clinical findings that TCAs are associated with an increase in the fracture rat via actions on the noradrenergic system. Thus, TCAs reduce sympathetic outflow significantly on one hand, while serving as norepinephrine reuptake blockers on the other. Moreover, even if tricyclics did increase availability of NE at receptors located on bone, their effects are so numerous that this effect alone might not necessarily lead to pathologic bone loss.

### Despite responsiveness to monoaminergic inputs, loss of BMD and fractures may not be related to antidepressants

We have previously noted that even young, premenopausal women with major depression have significant losses in BMD greater at the hip than at the spine. Many other studies find reduced BMD in both male and female populations of patients with depressive illness (for example, 7–9). The etiology of this loss of BMD is not known, but, as noted earlier, is likely to represent many factors, including a proinflammatory state, which is associated with preferential loss of bone at the hip compared with vertebral loss. The activation of the HPA axis and sympathetic nervous system, as well as inhibition of the growth hormone and gonadal axes could also contribute.

The data presented here suggest that antidepressants do not contribute to bone fractures after minimal trauma in older patients as these drugs do not reduce bone strength. Thus, increased fracture in older patients on antidepressants is likely to reflect factors intrinsic to depression itself. Accordingly, more older depressed patients will be on antidepressants than non-depressed patients, and hence, the fracture rate will be greater in patients on antidepressant treatment. Thus, preclinical studies showing that serotonin systems exist within bone and that bone is responsive to noradrenergic input do not, in themselves, implicate antidepressants in the diathesis to bone fracture in older depressed patients.

### Clinical implications: relationship between bone density, bone strength and susceptibility to fracture—who should be watched carefully

The assumption that BMD equates with bone strength and resistance to fracture has not been fully validated, especially in younger individuals.^26^ Rather, resistance to fracture is a composite of its microarchitecture, accumulated microscopic damage and the quality of collagen, mineral crystal size and bone turnover. Many lines of evidence challenge the generally accepted orthodoxy that bone density is the best way to assess strength of bone. Indeed, denser bone is not always stronger. This discrepancy came to light with the use of sodium fluoride to treat osteoporosis. Although sodium fluoride produced large increases in density, fluoride made the bone more brittle because it changed the quality of the mineral and rendered it more susceptible to fracturing, especially in vertebral fracture regions. Thus BMD cannot always be considered a straightforward surrogate marker for bone strength and resistance to fracture,^26^ though the two are likely to be related.

Young bone and older bone differ in strength, even with similar bone density. Hence, young bone is stronger than older bone across all levels of bone mass. Moreover, clinical studies showed that the drugs approved for treating osteoporotic fractures generally fare better than we would expect from their effects on bone density.^27^ Thus, bones become stronger before they grow more dense. These data suggest that premenopausal women with loss of BMD are not necessarily more susceptible to fracture because of other factors associated with younger age such as high quality collagen. However, they are losing BMD faster than non-depressed controls and should be followed closely after menopause, when vulnerability to fracture increases because of loss of strength and of estrogen.

Like BMD, bone strength decreases progressively as a function of age. In older individuals, decreased bone strength increases fracture risk. As BMD loss is greater in premenopausal women at the hip than at the spine, fractures may be a particular problem for older women who suffer from depression, a premise validated by clinical studies. Almost all of the papers cited earlier about antidepressant use and fractures in older women specifically indicated a preferential effect on hip fractures over fractures of the spine.^4, 5^

### Anticonvulsants

Anticonvulsant drugs such as CBZ and VAL are well known to affect BMD by altering the biochemical and mineral composition of bone tissue, and have been associated with a higher predisposition to bone fracture.^28^ These have been hypothesized to be caused by a vitamin D deficiency similar to osteomalacia.^19^ A recent study suggested that decreased BMD in patients treated with VAL might also be associated with increased bone resorption. VAL and CBZ may also interact with glucocorticoids in promoting bone loss.^29^

In contrast to antidepressants, we report here that anticonvulsant treatments have a substantial effect on mechanical and geometrical properties of bone (Table 2). The detrimental effects of CBZ on mechanical properties of bone are paralleled by a similar alteration in moment of inertia, indicating that this drug affects both the geometry of bone and its material properties. In contrast, decreases in mechanical properties caused by VAL were not associated with concomitant changes in cross-sectional geometry. Therefore, this drug treatment is likely to exert its effect on bone properties through changes in material properties of the bone tissue and not geometrical alterations. Previous clinical studies on the effects of VAL and CBZ on BMD in humans reported that VAL monotherapy, but not CBZ, significantly reduces axial and appendicular BMD.^18^ This indicates that material property alterations in VAL-treated animals may be related, in part, to changes in mineralization. These results portray a potentially important association between anticonvulsant therapy and bone mass, and may reveal a novel mechanism affecting its regulation.

Anticonvulsants such as VAL and CBZ are often used not only in patients with epilepsy, but also in patients with bipolar disorder. Despite the fact that BMD and bone strength do not always correlate, BMD should be followed in depressed patients on anticonvulsants, as bone strength is likely to correlate with BMD, at least partially. The combination of depressive illness, anticonvulsants and old age may be particularly toxic to bone health.

## Figures and Tables

**Table 1:** Geometrical properties of antidepressant- and anticonvulsant-treated rats

	*CSA (mm^2^)*	*CT (mm)*	*Ixx (mm^4^)*
IM (*n*=15)	6.27±0.49	0.696±0.052	9.54±1.35
FL (*n*=12)	6.48±0.50	0.725±0.052	9.75±1.67
VA (*n*=9)	6.36±0.36	0.740±0.095	9.32±0.94
SA (*n*=12)	6.55±0.34	0.715±0.026	10.10±0.34
CA (*n*=9)	5.53±0.46**	0.640±0.044*	7.78±1.03**
CC (*n*=11)	6.23±0.33	0.671±0.020	9.52±1.24

Abbreviations: CA, carbamazepine; CC, controls; CSA, cross-sectional area; CT, cortical thickness; FL, fluoxetine; IM, imipramine; Ixx, moment of inertia along x-x axis; SA, saline; VA, valproate.

All values expressed as mean±s.d.

^*^P<0.05 vs CC.

^**^P<0.005 vs CC.

**Table 2:** General effects

	*Antidepressants*		*Anticonvulsants*		*Sham treatment*	
	*Imipramine*	*Fluoxetine*	*Valproate (VAL)*	*Carbama (CBZ)*	*Saline (SAL)*	*Carbama Ctrl*
Stiffness			↓ 18.5%^a^	↓ 21%^b^		
Yield			↓ 25.3%	↓ 26%		
Moment of inertia			↓ 2.2%	↓ 26.3%		
Stiffness						
Geometrical changes				Yes		

a Compared with saline.

b Compared with carbamazepine control.

Anticonvulsants decreased the mechanical properties of the whole bone. Both valproic acid (VAL) and carbamazepine (carbama) reduced stiffness (18.5% and 21.3% compared with saline and carbamazepine controls, respectively) and yield (25.3% and 26.4% compared with saline and carbamazepine controls, respectively). Alterations in mechanical properties were accompanied by geometrical changes (26.3% reduction in the moment of inertia) in the carbamazepine group only. No significant changes in the effects of antidepressants.

## Appendix

The moment of inertia of an object about a given axis describes how difficult it is to change its angular motion about that axis. Therefore, it encompasses not just how much mass the object has overall, but how far each bit of mass is from the axis. The further out the object's mass is, the more rotational inertia the object has, and the more torque (force × distance from axis of rotation) is required to change its rotation rate. For example, consider two hoops, A and B, made of the same material and of equal mass. Hoop A is larger in diameter but thinner than B. It requires more effort to accelerate hoop A (change its angular velocity) because its mass is distributed farther from its axis of rotation: mass that is farther out from that axis must, for a given angular velocity, move more quickly than mass closer in. So in this case, hoop A has a larger moment of inertia than hoop B.
